# Inertial Sensors—Applications and Challenges in a Nutshell

**DOI:** 10.3390/s20216221

**Published:** 2020-10-31

**Authors:** Thomas Seel, Manon Kok, Ryan S. McGinnis

**Affiliations:** 1 Control Systems Group, Technische Universität Berlin, 10587 Berlin, Germany; 2 Delft Center for Systems and Control, Delft University of Technology, 2628 CD Delft, The Netherlands; m.kok-1@tudelft.nl; 3 Department of Electrical and Biomedical Engineering, University of Vermont, Burlington, VT 05405, USA; Ryan.McGinnis@uvm.edu

**Keywords:** accelerometers, gyroscopes, magnetometers, sensor fusion, body area networks, inertial motion tracking, error modeling and calibration, localization and mapping, machine learning applied to inertial sensor data, inertial sensors in vehicle motion estimation and control, inertial sensors in robotics and manufacturing, inertial sensors in health care and sports engineering

## Abstract

This editorial provides a concise introduction to the methods and applications of inertial sensors. We briefly describe the main characteristics of inertial sensors and highlight the broad range of applications as well as the methodological challenges. Finally, for the reader’s guidance, we give a succinct overview of the papers included in this special issue.

Inertial sensors have become a key enabling technology in a wide variety of applications, and are currently found in almost every digital device and intelligent vehicle. Inertial sensor information is, for instance, used to facilitate localization, navigation, and mapping [1,2,3,4,5,6]. Wearable inertial sensors are also frequently used in health care and sporting applications for capturing movement patterns outside of typical laboratory environments [7,8,9,10].

Most of the large number of new applications strongly benefit from the hardware miniaturization and advances in micro-electromechanical systems that have led to sensor designs with much smaller dimensions, energy demands, weights, and costs [9,11]. However, despite these decisive advantages, inertial sensors impose several substantial signal processing challenges, which we briefly summarize in the following. 

The measurements of an inertial measurement unit (IMU) are obtained in the moving coordinate system of the sensor, which complicates their time integration and direct interpretation [12]. Three-dimensional strapdown integration is required, as well as suitable sensor fusion methods, since the measured angular rates, accelerations and magnetic field vectors rarely coincide with the actual motion states of interest, such as orientations, velocities or joint angles, or even more high-level parameters. Moreover, the sensor coordinate systems are often not sufficiently well-aligned with a meaningful coordinate system of the object of interest, to which the sensor is attached [13,14]. Furthermore, especially in biomechanics, this connection is often not completely rigid [5]. This has led to a large interest in methods for non-restrictive sensor-to-segment calibration or anatomical calibration on the one hand and soft-tissue motion artifact compensation on the other hand [6,15].

Magnetic disturbances are another major challenge that frequently appear in wearable systems and other applications, in particular in indoor environments. The magnetic field inside buildings and near electronic devices or ferromagnetic material is known to be inhomogeneous to the extent that magnetometer measurements no longer yield accurate heading information [16]. Although these inhomogeneities have been exploited to facilitate indoor navigation [17,18,19], they complicate the estimation of absolute orientations [20,21,22] and complete motion states in kinematic chains [23,24,25,26], and necessitate the use of additional models or measurement information. 

Finally, and likewise notably, the measurement signals of inertial sensors are well-known to be corrupted by errors such as time-variant sensor biases and measurement noise [27]. Therefore, pure strap-down integration of the sensor signals to orientations and positions yields substantial integration drift and erroneous results [12]. This challenge is commonly addressed by combining inertial measurements with complementary information provided by, for example, additional sensor measurements or mathematical models [28,29]. This explains why, much more than for most other sensors, the use of inertial measurement units entails the employment of advanced sensor fusion methods, kinematic models, mathematical methods and algorithms. 

In recent years, advanced sensor fusion and state estimation methods have been developed to combine this information and minimize the influence of the sensor errors on the motion states of interest. Amongst these are filtering approaches, such as extended and unscented Kalman filters and complementary filters, which enable real-time estimation. Offline smoothing approaches have also been developed that allow the use of all recorded data to improve estimation performance. 

In human motion tracking, it has become increasingly common to include additional information from kinematic models to aid the estimation. Including these models overcomes the need for additional measurement modalities (e.g., magnetometer measurements) to estimate the relative orientation of different body segments [23,24,25,26]. Furthermore, promising new directions of research focus on the use of sparse sensor setups, where fewer inertial sensors are placed on the body than the number of body segments of interest [30]. 

Machine learning methods have also emerged as a promising new direction. These methods can be used both for classification and regression. For instance, they can be used to classify human activities [7], detect disease [31] or risk status [32], or estimate continuous variables like walking speed [33] when deployed on inertial sensor data. They can also be used to learn the orientation of the sensor [34], for vehicle motion estimation [35] and for localization and mapping [36].

The papers in this special issue cover a large variety of the outlined topics and recent progress in the methods and applications of inertial sensors. Both model-based and data-based approaches are considered on the methodological side, while the covered fields of applications include inertial navigation [37], robotics [38,39,40], and the use of wearable inertial sensors for efficiently capturing human biomechanics, gestures, and activities [41,42,43,44,45,46,47,48,49,50]. 

Three of the papers in this special issue find their motivation in the area of robotics, albeit from diverse perspectives. Problems tackled include orientation estimation for mobile robots [38], a control scheme for stabilizing optical payloads in unmanned aerial vehicles [40], and trajectory estimation and mapping for ground robots [39]. Each of these topics target important engineering goals that enhance the performance of these robots and leverage the complementary data sources inherent to all inertial sensors. 

Ten of the papers in this special issue present advances in the use of wearable inertial sensors for efficiently capturing human biomechanics, gestures and activities. Motivations for these studies include enabling novel assessment and intervention, reducing communication loads for wireless sensor networks, and improving human–computer interaction. Notably, several of the papers present new approaches for estimating knee [43] and hip [44] joint angles, and knee joint forces [47] that are validated in human subjects. The use of wearable inertial sensors for estimating human joint kinematics is also explored in a systematic review [46]. These studies are important, as they add to the body of evidence demonstrating the ability of wearable inertial sensors to provide objective measurements of biomechanical quantities important for characterizing human health.

In summary, this special issue presents a broad collection of current research contributions to the rapidly evolving field of inertial sensor methods and applications. Many of the presented works and future works that will build thereon can be expected to help solve the aforementioned challenges and contribute to the further spread and establishment of inertial sensor technologies in a wide range of application domains.
